# Supplementary material for: A UMLS-based spell checker for natural language processing in vaccine safety
Source: BMC Med Inform Decis Mak. 2007 Feb 12;7:3. doi: 10.1186/1472-6947-7-3 (PMC1805499; doi:10.1186/1472-6947-7-3)
Supplement: Additional file 13 — RAP application directory. Sets up directory for RAP (RDF application for PHP) source code [file 1472-6947-7-3-S13.gz › rap/doc/phpdoc/__filesource/fsource_model__modelStatement.php.html]

xml version="1.0" encoding="iso-8859-1"?


File Source for Statement.php


# Source for file Statement.php

Documentation is available at Statement.php

```
1. <?php

3. // ----------------------------------------------------------------------------------
4. // Class: Statement
5. // ----------------------------------------------------------------------------------


9. /**
10. * An RDF statement.
11. * In this implementation, a statement is not itself a resource.
12. * If you want to use a a statement as subject or object of other statements,
13. * you have to reify it first.
14. *
15. * <BR><BR>History:<UL>
16. * <LI>05-30-2003                : Changed function compare(), added statementsorter($a,$b).</LI>
17. * <LI>02-21-2003                : getLabelSubject(), getLabelPredicate(), getLabelObject(), toStringSubject(), toStringPredicate(), toStringObject() added.</LI>
18. * <LI>09-10-2002                : First version of this class.</LI>
19. *
20. * @author Chris Bizer <chris@bizer.de>
21. * @version  V0.9.1
22. * @package model
23. */
24. class Statement extends Object {

26. /**
27. * Subject of the statement
28. *
29. * @var        object resource
30. * @access    private
31. */
32. var $subj;

34. /**
35. * Predicate of the statement
36. *
37. * @var        object resource
38. * @access    private
39. */
40. var $pred;

42. /**
43. * Object of the statement
44. *
45. * @var        object node
46. * @access    private
47. */
48. var $obj;

50. /**
51. * The parameters to constructor are instances of classes and not just strings
52. *
53. * @param    object    node $subj
54. * @param    object    node $pred
55. * @param    object    node $obj
56. * @throws    PhpError
57. */
58. function Statement($subj, $pred, $obj) {

60. if (!is_a($subj, 'Resource')) {
61. $errmsg = RDFAPI_ERROR .
62. '(class: Statement; method: new): Resource expected as subject.';
63. trigger_error($errmsg, E_USER_ERROR);
64. }
65. if (!is_a($pred, 'Resource') || is_a($pred, 'BlankNode')) {
66. $errmsg = RDFAPI_ERROR .
67. '(class: Statement; method: new): Resource expected as predicate, no blank node allowed.';
68. trigger_error($errmsg, E_USER_ERROR);
69. }
70. if (!(is_a($obj, 'Resource') or is_a($obj, 'Literal'))) {
71. $errmsg = RDFAPI_ERROR .
72. '(class: Statement; method: new): Resource or Literal expected as object.';
73. trigger_error($errmsg, E_USER_ERROR);
74. }

76. $this->pred = $pred;
77. $this->subj = $subj;
78. $this->obj = $obj;
79. }

81. /**
82. * Returns the subject of the triple.
83. * @access    public
84. * @return    object node
85. */
86. function getSubject() {
87. return $this->subj;
88. }

90. /**
91. * Returns the predicate of the triple.
92. * @access    public
93. * @return    object node
94. */
95. function getPredicate() {
96. return $this->pred;
97. }

99. /**
100. * Returns the object of the triple.
101. * @access    public
102. * @return    object node
103. */
104. function getObject() {
105. return $this->obj;
106. }

108. /**
109. * Alias for getSubject()
110. * @access    public
111. * @return    object node
112. */
113. function subject() {
114. return $this->subj;
115. }

117. /**
118. * Alias for getPredicate()
119. * @access    public
120. * @return    object node
121. */
122. function predicate() {
123. return $this->pred;
124. }

126. /**
127. * Alias for getObject()
128. * @access    public
129. * @return object node
130. */
131. function object() {
132. return $this->obj;
133. }
134. /**
135. * Retruns the hash code of the triple.
136. * @access    public
137. * @return string
138. */
139. function hashCode()  {
140. return md5($this->subj->getLabel() . $this->pred->getLabel()  . $this->obj->getLabel());
141. }

143. /**
144. * Dumps the triple.
145. * @access    public
146. * @return string
147. */

149. function toString() {
150. return  'Triple(' . $this->subj->toString() . ', ' . $this->pred->toString() . ', ' . $this->obj->toString() . ')';

152. }

154. /**
155. * Returns a toString() serialization of the statements's subject.
156. *
157. * @access    public
158. * @return    string
159. */
160. function toStringSubject() {
161. return $this->subj->toString();
162. }

164. /**
165. * Returns a toString() serialization of the statements's predicate.
166. *
167. * @access    public
168. * @return    string
169. */
170. function toStringPredicate() {
171. return $this->pred->toString();
172. }

174. /**
175. * Reurns a toString() serialization of the statements's object.
176. *
177. * @access    public
178. * @return    string
179. */
180. function toStringObject() {
181. return $this->obj->toString();
182. }

184. /**
185. * Returns the URI or bNode identifier of the statements's subject.
186. *
187. * @access    public
188. * @return    string
189. */
190. function getLabelSubject() {
191. return $this->subj->getLabel();
192. }

194. /**
195. * Returns the URI of the statements's predicate.
196. *
197. * @access    public
198. * @return    string
199. */
200. function getLabelPredicate() {
201. return $this->pred->getLabel();
202. }

204. /**
205. * Reurns the URI, text or bNode identifier of the statements's object.
206. *
207. * @access    public
208. * @return    string
209. */
210. function getLabelObject() {
211. return $this->obj->getLabel();
212. }

214. /**
215. * Checks if two statements are equal.
216. * Two statements are considered to be equal if they have the
217. * same subject, predicate and object. A statement can only be equal
218. * to another statement object.
219. * @access    public
220. * @param        object    statement $that
221. * @return    boolean
222. */

224. function equals ($that) {

226. if ($this == $that) {
227. return true;
228. }
229. if ($that == NULL || !(is_a($that, 'Statement'))) {
230. return false;
231. }

233. return
234. $this->subj->equals($that->subject()) &&
235. $this->pred->equals($that->predicate()) &&
236. $this->obj->equals($that->object());
237. }

239. /**
240. * Compares two statements and returns integer less than, equal to, or greater than zero.
241. * Can be used for writing sorting function for models or with the PHP function usort().
242. *
243. * @access    public
244. * @param        object    statement &$that
245. * @return    boolean
246. */

248. function compare(&$that) {
249. return statementsorter($this, $that);
250. // statementsorter function see below
251. }


254. /**
255. * Reifies a statement.
256. * Returns a new MemModel that is the reification of the statement.
257. * For naming the statement's bNode a Model or bNodeID must be passed to the method.
258. *
259. * @access    public
260. * @param        mixed    &$model_or_bNodeID
261. * @return    object    model
262. */

264. function & reify(&$model_or_bNodeID) {

266. if (is_a($model_or_bNodeID, 'MemModel')) {
267. // parameter is model
268. $statementModel = new MemModel($model_or_bNodeID->getBaseURI());
269. $thisStatement = new BlankNode($model_or_bNodeID);
270. } else {
271. // parameter is bNodeID
272. $statementModel = new MemModel();
273. $thisStatement = new BlankNode($model_or_bNodeID);
274. }

276. $RDFstatement = new Resource(RDF_NAMESPACE_URI . RDF_STATEMENT);
277. $RDFtype = new Resource(RDF_NAMESPACE_URI . RDF_TYPE);
278. $RDFsubject = new Resource(RDF_NAMESPACE_URI . RDF_SUBJECT);
279. $RDFpredicate = new Resource(RDF_NAMESPACE_URI . RDF_PREDICATE);
280. $RDFobject = new Resource(RDF_NAMESPACE_URI . RDF_OBJECT);

282. $statementModel->add(new Statement($thisStatement, $RDFtype, $RDFstatement));
283. $statementModel->add(new Statement($thisStatement, $RDFsubject, $this->getSubject()));
284. $statementModel->add(new Statement($thisStatement, $RDFpredicate, $this->getPredicate()));
285. $statementModel->add(new Statement($thisStatement, $RDFobject, $this->Object()));

287. return $statementModel;
288. }

290. } // end: Statement


293. /**
294. * Comparison function for comparing two statements.
295. * statementsorter() is used by the PHP function usort ( array array, callback cmp_function)
296. *
297. * @access    private
298. * @param        object Statement    $a
299. * @param        object Statement    $b
300. * @return    integer less than, equal to, or greater than zero
301. * @throws phpErrpr
302. */
303. function statementsorter($a,$b) {
304. //Compare subjects
305. $x=$a->getSubject();
306. $y=$b->getSubject();
307. $r=strcmp($x->getLabel(),$y->getLabel());
308. if ($r!=0) return $r;
309. //Compare predicates
310. $x=$a->getPredicate();
311. $y=$b->getPredicate();
312. $r=strcmp($x->getURI(),$y->getURI());
313. if ($r!=0) return $r;
314. //Final resort, compare objects
315. $x=$a->getObject();
316. $y=$b->getObject();
317. return strcmp($x->toString(),$y->toString());
318. }

320. ?>
```

Documentation generated on Thu, 7 Jul 2005 13:43:56 +0200 by phpDocumentor 1.3.0RC3
